# Oct4 transcriptionally regulates the expression of long non-coding RNAs *NEAT1* and *MALAT1* to promote lung cancer progression

**DOI:** 10.1186/s12943-017-0674-z

**Published:** 2017-06-14

**Authors:** Jayu Jen, Yen-An Tang, Ying-Hung Lu, Che-Chung Lin, Wu-Wei Lai, Yi-Ching Wang

**Affiliations:** 1 Department of Pharmacology, College of Medicine, National Cheng Kung University, Tainan, 701 Taiwan; 2 Institute of Basic Medical Sciences, College of Medicine, National Cheng Kung University, Tainan, 701 Taiwan; 3 Division of Thoracic Surgery, Department of Surgery, National Cheng Kung University Hospital, College of Medicine, National Cheng Kung University, Tainan, 701 Taiwan

**Keywords:** Oct4, lncRNA, *MALAT1*, *NEAT1*, Transcription regulation, Lung cancer

## Abstract

**Background:**

Oct4, a key stemness transcription factor, is overexpressed in lung cancer. Here, we reveal a novel transcription regulation of long non-coding RNAs (lncRNAs) by Oct4. LncRNAs have emerged as important players in cancer progression.

**Methods:**

Oct4 chromatin-immunoprecipitation (ChIP)-sequencing and several lncRNA databases with literature annotation were integrated to identify Oct4-regulated lncRNAs. Luciferase activity, qRT-PCR and ChIP-PCR assays were conducted to examine transcription regulation of lncRNAs by Oct4. Reconstitution experiments of Oct4 and downstream lncRNAs in cell proliferation, migration and invasion assays were performed to confirm the Oct4-lncRNAs signaling axes in promoting lung cancer cell growth and motility. The expression correlations between Oct4 and lncRNAs were investigated in 124 lung cancer patients using qRT-PCR analysis. The clinical significance of Oct4/lncRNAs signaling axes were further evaluated using multivariate Cox regression and Kaplan-Meier analyses.

**Results:**

We confirmed that seven lncRNAs were upregulated by direct binding of Oct4. Among them, *nuclear paraspeckle assembly transcript 1* (*NEAT1*), *metastasis-associated lung adenocarcinoma transcript 1* (*MALAT1*) and *urothelial carcinoma-associated 1* (*UCA1*) were validated as Oct4 transcriptional targets through promoter or enhancer activation. We showed that lung cancer cells overexpressing *NEAT1* or *MALAT1* and the Oct4-silenced cells reconstituted with *NEAT1* or *MALAT1* promoted cell proliferation, migration and invasion. In addition, knockdown of *NEAT1* or *MALAT1* abolished Oct4-mediated lung cancer cell growth and motility. These cell-based results suggested that Oct4/*NEAT1* or Oct4/*MALAT1* axis promoted oncogenesis*.* Clinically, Oct4/*NEAT1*/*MALAT1* co-overexpression was an independent factor for prediction of poor outcome in 124 lung cancer patients.

**Conclusions:**

Our study reveals a novel mechanism by which Oct4 transcriptionally activates *NEAT1* via promoter and *MALAT1* via enhancer binding to promote cell proliferation and motility, and led to lung tumorigenesis and poor prognosis.

**Electronic supplementary material:**

The online version of this article (doi:10.1186/s12943-017-0674-z) contains supplementary material, which is available to authorized users.

## Background

Oct4, encoded by *POU5F1* (POU domain, class 5, transcription factor 1), is a homeodomain transcription factor of the POU family. Oct4, Sox2 and Nanog are well-known pluripotency-associated transcription factors which maintain embryonic stem cells state [1]. Metaplastic transformation, a precancerous condition, has been reported to recapitulate embryonic development. Therefore, the key factors involved in embryonic development may play critical roles in carcinogenesis. Studies have shown that Oct4 is overexpressed in human cancers such as bladder [2], breast [3], cervical cancer [4], oral squamous cell carcinoma [5], hepatocellular carcinoma [6] and lung cancer [7, 8]. In embryonic stem cells, Oct4 has been identified to regulate transcriptions of other transcription factors, chromatin modifiers, long non-coding RNAs (lncRNAs) and microRNAs [9, 10]. For instance, Oct4 regulates lncRNAs expression, such as linc-RoR, which is a key reprogramming factor associated with pluripotency [11]. Oct4 can also interact with Pontin, a chromatin remodeling factor, to regulate the transcription of lncRNAs, including linc1253, a lineage programme repressing lincRNA [12]. However, transcription regulation of lncRNAs by Oct4 in tumorigenesis remains elusive.

LncRNAs is a subset of non-coding RNAs with length ranging from 200 nucleotides to 100,000 nucleotides. According to data obtained using next generation RNA-sequencing, the number of total human lncRNAs is approximately 20,000 transcripts and over 200 lncRNAs are confirmed to be functional [13, 14]. Some lncRNAs are dysregulated in cancers and may serve as potential prognostic markers for specific cancer types [15, 16]. Some lncRNAs have been characterized to possess oncogene-like or tumor suppressor-like function. For instance, *Hox Antisense Intergenic RNA (HOTAIR)*, acts as a bridge between PRC2 chromatin repressive and LSD1/CoREST/REST corepressor complexes to further modulate the metastasis-related gene expressions through changing chromatin states in breast cancer [15, 17]. Another lncRNA, *HOXA transcript at the distal tip (HOTTIP)*, not only promotes pancreatic cancer progression but also confers chemoresistance to gemcitabine, which may be mediated by HOXA13 [18, 19]. Accumulating evidence indicates that lncRNAs play critical roles in cancer biology.

Up to date, most of the studies on lncRNAs focus on the outcome and underlying mechanisms of dysregulated lncRNAs and their potential as prognosis markers. However, little is known about the upstream regulations responsible for aberrant expression of lncRNAs in cancers, especially at the transcriptional level. Our previous study using chromatin-immunoprecipitation sequencing (ChIP-seq) and functional analyses revealed a critical Oct4-driven transcriptional program [8]. Genome-wide analysis of Oct4 targeting of this program suggests a novel role of Oct4-mediated transcriptional regulation of lncRNAs. In the current study, we have shown that Oct4 transcriptionally activated oncogenic lncRNAs expression through promoter- or enhancer-binding regulation. Moreover, Oct4-mediated high expression of lncRNAs such as *nuclear paraspeckle assembly transcript 1* (*NEAT1*) and *metastasis-associated lung adenocarcinoma transcript 1* (*MALAT1*) promoted lung cancer cell proliferation, migration and invasion abilities. Clinical studies further validated the importance of Oct4/*NEAT1*/*MALAT1* signaling axis in lung cancer progression.

## Methods

### Cell lines and culture conditions

Human lung adenocarcinoma cell line A549 and normal bronchial epithelial cell line BEAS-2B was purchased from American Tissue Culture Company (ATCC). Human lung adenocarcinoma cell line CL1–0 was obtained from Dr. Pan-Chyr Yang (Department of Internal Medicine Medical College, National Taiwan University, Taiwan). All media were supplemented with 10% Fetal Bovine Serum (Gibco, Carlsbad, CA, USA) and 1% penicillin/streptomycin (Gibco). Stable cell line expressing Oct4 or empty vector was established by ectopic transfection of Flag-Oct4 or empty vector plasmid into A549 and CL1–0 cells, and selected with puromycin. Transient transfections of Oct4 in BEAS-2B were carried out with lipofetamine 2000 (Invitrogen, Carslbad, CA, USA).

### Transfection of plasmids and RNAi

The plasmids used in the study are listed in Additional file 1: Table S1. The interference RNA (RNAi) for Oct4 was obtained from Invitrogen (# Oct4-HSS143403, Invitrogen). Depletion of *NEAT1* or *MALAT1* was performed by transfection of smart-pool siRNAs (Dharmacon, Lafayette, CO, USA) at final concentration of 10 nM. Transfections of expression plasmids and RNAi were performed using lipofectamine 2000 (Invitrogen, Carslbad, CA, USA) according to the manufacturer’s protocol.

### Chromatin-immunoprecipitation-polymerase chain reaction (ChIP-PCR) assay

Empty vector control and Oct4 stably-overexpressed A549 cells (1 × 10^7^ cells) were cross-linked with 1% formaldehyde for 10 min at 37 °C, followed by preparation of nuclear lysates using Magna ChIP™ protein G Kit (Millipore Co., Billerica, MA, USA). Nuclear lysates were sonicated to shear crosslinked DNA to around 300 ~ 500 bps using Covaris-S2 machine. Chromatin was immunoprecipitated with Oct4 antibody (1:100, # ab-19857, Abcam, Cambridge, UK). Purified chromatin-immunoprecipitated DNA was subjected to PCR analysis using primers for the lncRNA promoter and enhancer regions listed in Additional file 1: Table S2.

### RNA extraction and quantitative reverse transcriptase-polymerase chain reaction (qRT-PCR) assays

Four μg of total RNA was reverse transcribed to cDNA using MultiScribe™ reverse transcriptase (Applied Biosystems, Foster City, CA, USA). cDNA was amplified using the Fast SYBR® Green Master Mix (Applied Biosystems). qRT-PCR was used to measure *Oct4* mRNA and lncRNA expression using the StepOnePlus™ Real-Time PCR System (Applied Biosystems). The primer sequences and annealing temperature are listed in Additional file 1: Table S3.

### Site-directed mutagenesis and luciferase promoter/enhancer activity assays

Mutations (Mut) of Oct4 binding elements within the *NEAT1* promoter, *MALAT1* enhancer or *urothelial carcinoma-associated 1 (UCA1)* enhancer were generated by site-directed mutagenesis using wild-type (WT) *NEAT1* promoter, *MALAT1* enhancer or *UCA1* enhancer vectors as templates. The primers used are described in Additional file 1: Table S4.

For luciferase activity assays, cells were seeded the day before transfection. The pGL4-Renilla construct was included as an internal control. After 16 h of co-transfection with empty vector or gene promoter/enhancer vector, and pGL3-Basic or pGL4-Renilla, the dual luciferase reporter assay kit (Promega, Madison, WI, USA) was used to determine gene promoter or enhancer activity. The luminescence was measured with a Turner BioSystems luminometer (Promega). The data are represented as the means of ratio of firefly luciferase to Renilla luciferase activity by triplicate experiments.

### RealTime-Glo viability assay

Cell viability was assayed using RealTime-Glo assay (Promega). Briefly, cells were transfected for 24 h and then reseeded at 2 × 10^3^ cells/well in 96-well plates. MT Cell Viability Substrate and NanoLuc Enzyme were diluted and added to each well. The luminescence was measured with a Turner BioSystems luminometer (Promega) at 24, 48 and 72 h.

### Transwell migration and invasion assay

The transwell insert with millipore membrane (pore size of 8 μm, Falcon, BD Franklin Lakes, NJ, USA) was used. For transwell migration assay, 2 × 10^5^ A549 cells and 5 × 10^5^ CL1–0 cells were seeded onto the upper chamber with 1 ml serum-free medium. For transwell invasion assay, the transwell inserted membranes were pre-coated with Matri-gel (2.5 mg/ml, Sigma-Aldrich, St. Louis, MO, USA) 1 day before seeding cells. Complete medium with 20% FBS was supplemented into the lower chamber as chemoattractants. The cells were incubated for 16 ~ 24 h and then the cells attached on the reverse side of the membrane were then fixed and stained. Six random views were photographed and quantified under an upright microscope (Nikon E400, Yurakucho, Tokyo, Japan).

### Study population

We recruited 124 lung cancer patients from National Cheng Kung University Hospital after obtaining appropriate institutional review board permission and informed consent from the patients. Surgically resected tumor tissue and corresponding normal tissue samples were collected. Total RNA of patient samples were prepared using Trizol reagent (Invitrogen) and reverse transcribed into cDNA as described above. qRT-PCR was conducted to measure the expressions of *Oct4*, *NEAT1* and *MALAT1* using the StepOnePlus™ Real-Time PCR System (Applied Biosystems). The expression of the target genes was normalized based on the levels of internal control gene, *GAPDH*. The primers used for qRT-PCR analyses are described in Additional file 1: Table S3.

### Statistical analysis

Pearson χ^2^ test was used to compare the correlation of Oct4 and lncRNAs expression and clinicopathological parameters in lung cancer patients. Overall survival curves were calculated according to the Kaplan-Meier method, and comparison was performed using the log-rank test. Two-way ANOVA and two-tailed Student’s *t*-test was used in cell and animal studies. Data represent mean ± SEM. *P* < 0.05 was considered to be statistically significant.

## Results

### Unbiased ChIP-seq and ChIP-PCR/qRT-PCR analyses reveal novel lncRNAs controlled by Oct4 transcriptional regulation in lung cancer

**Fig. 1:**
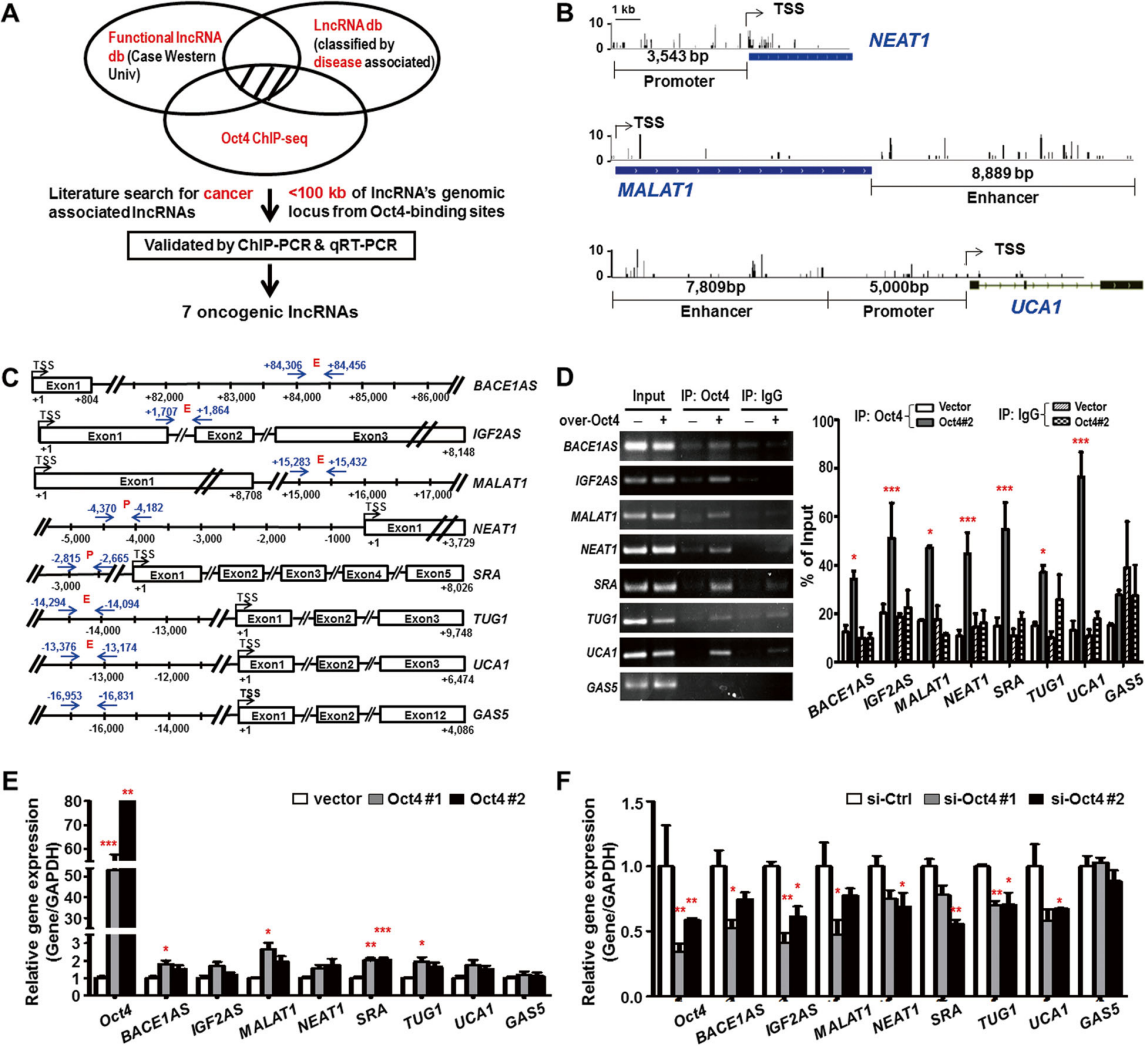
Oncogenic lncRNAs were revealed as Oct4 transcriptional downstream targets in lung cancer using ChIP-seq, ChIP-PCR and qRT-PCR assays. **a** Flowchart of strategies used to identify Oct4 targeted downstream lncRNAs. **b** The Oct4 binding peaks at promoter and enhancer regions of three representative lncRNAs *NEAT1*, *MALAT1* and *UCA1*. **c** Schematic diagram depicting the ChIP-PCR primers for amplification of the regions including Oct4 binding sites around the eight lncRNAs genomic locus (indicated as *blue arrows*). TSS: transcription start site as indicated by (+1). Oct4 binding regions were classified as enhancer (E) or promoter (P). **d** ChIP-PCR analysis of Oct4 occupancy at the binding sites of the eight lncRNAs genomic loci. *GAS5* serves as a negative control lncRNA. Results are normalized to input by semi-quantitative analysis. IgG serves as an experimental negative control. Data represent mean ± SEM. *P*-values were determined by two-way ANOVA. **e**, **f** qRT-PCR analysis of eight lncRNAs expressions in A549 cells stably overexpressing Oct4 (Oct4#1, Oct4#2) (**e**) or Oct4-silenced A549 cells (si-Oct4#1, si-Oct4#2) (**f**). *GAS5* serves as a negative control lncRNA. Target lncRNA expression levels were normalized to *GAPDH* expression levels. Data represent mean ± SEM. *P*-values were determined by two-tailed Student *t*-test. *P < 0.05; **P < 0.01; ***P < 0.001

We previously performed ChIP-seq in A549 lung cancer cell line stably-overexpressing Oct4 to identify the Oct4 genome-wide DNA binding regions [8]. Notably, genomic loci of some lncRNAs were bound by Oct4. We further identified the potential Oct4-regulated lncRNAs through the following bioinformatic and functional analyses. Firstly, we integrated our in-house ChIP-seq dataset with functional lncRNA database [20] and lncRNA db [21] with literature annotation to search for lncRNAs with oncogenic potential. Secondly, candidate lncRNAs were further selected following the criteria that the Oct4 binding sites located within 100 kb from lncRNAs genomic loci. We then performed ChIP-PCR and qRT-PCR in A549 and CL1–0 cells overexpressing Oct4 to validate the selected lncRNAs for Oct4 protein binding and transcriptional regulation, seven oncogenic lncRNAs were validated (Fig. 1a). A549 and CL1–0 lung cancer cells were confirmed for the Oct4-induced oncogenic effects in vitro and in vivo (Additional file 1: Figure S1). Visualization of Oct4 ChIP-seq targeting revealed significant enrichments of Oct4 binding on three lncRNAs, including the promoter region of *NEAT1* and enhancer regions of *MALAT1* and *UCA1* (Fig. 1b).

To validate ChIP-seq data, we performed ChIP-PCR and qRT-PCR to elucidate whether Oct4 transcriptionally regulates the eight selected lncRNAs, including seven oncogenic lncRNAs *BACE1AS*, *IGF2AS*, *MALAT1*, *NEAT1*, *SRA*, *TUG1* and *UCA1*, and tumor suppressor lncRNA *GAS5*. The *GAS5* was selected as a negative control lncRNA because it contained no ChIP-seq binding signal of Oct4. We used ALGGEN PROMO and TFSEARCH softwares to identify the putative Oct4 consensus binding elements (5’ATGCAAAT3’) of ChIP-seq regions. Oct4 binding sites within 5 kb upstream of transcription start site (TSS) were defined as promoter region, whereas Oct4 binding regions located at more than 5 kb of TSS or within the gene body were defined as enhancer region according to previous studies [22, 23]. Therefore, we classified the Oct4 binding sites to promoter (P) and enhancer (E) in relation to TSS of the seven lncRNAs (Fig. 1c). The ChIP-PCR results showed that Oct4 indeed targeted the predicted promoter or enhancer regions of the oncogenic lncRNAs but not the negative control lncRNA *GAS5* (Fig. 1d).

To further confirm the transcription activation of Oct4 on downstream lncRNAs, qRT-PCR analysis was conducted in both A549 and CL1–0 cells stably expressing Oct4. Results showed that overexpression of Oct4 in A549 increased the expression of the seven oncogenic lncRNAs but not that of the lncRNA *GAS5* (Fig. 1e). In contrast, knockdown of Oct4 in A549 cells decreased expression of the oncogenic lncRNAs but not that of *GAS5* (Fig. 1f). The results in CL1–0 cells showed a similar trend but to a lesser extent compared to that in A549 cells (Additional file 1: Figure S2). An increased expression of *NEAT1* and *UCA1* lncRNAs upon Oct4 overexpression was observed in the normal bronchial epithelial cell line BEAS-2B (Additional file 1: Figure S3). These results suggested that Oct4 may transcriptionally upregulate the expression of these oncogenic lncRNAs identified.

### Oct4 transcriptionally activates *NEAT1* via promoter and activates *MALAT1* and *UCA1* via enhancer binding

**Fig. 2:**
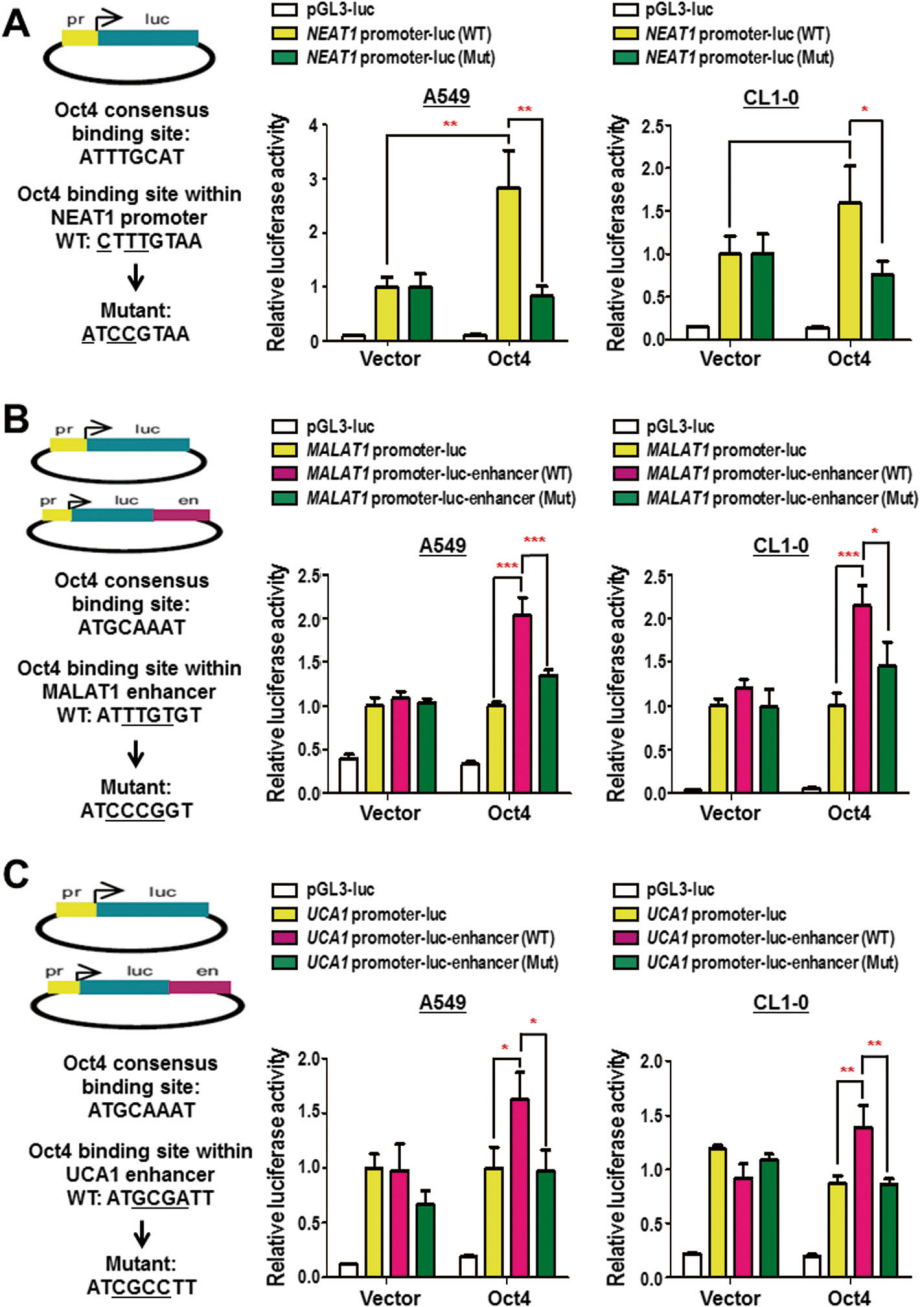
Oct4 promoted oncogenic lncRNAs transcription through activating promoter of *NEAT1* and enhancer of *MALAT1* and *UCA1*. **a** Schematic diagram depicting the construction of promoter activity assay of *NEAT1* (*upper left*). The wild-type (WT) and mutation (Mut) sites at the Oct4 consensus region are shown (*lower left*). Dual luciferase assay performed in A549 (*middle*) and CL1–0 (*right*) cells. **b**, **c** Schematic diagram depicting the construction of enhancer activity assay (*upper left*). The minimal promoters reported were inserted upstream of the luciferase reporter, while the Oct4 binding ChIP-seq regions were inserted downstream as the enhancer plasmids. The WT and Mut sites at the Oct4 consensus region are shown (*lower left*). Cells were transfected with vector or Oct4 plasmids and luciferase plasmids of *NEAT1* (**a**) *MALAT1* (**b**) or *UCA1* (**c**). Data are mean ± SEM. *P*-values were determined by two-way ANOVA. *P < 0.05; **P < 0.01; ***P < 0.001

To investigate the mechanism underlying Oct4-mediated lncRNAs upregulation, we selected *NEAT1*, *MALAT1* and *UCA1* lncRNAs for further transcriptional analyses. Oct4 was validated to bind at *NEAT1* promoter region while to *MALAT1* and *UCA1* at their enhancer regions (Fig. 1c and d). *NEAT1* promoter derived from the Oct4 binding ChIP-seq region was inserted upstream of the luciferase reporter gene (Fig. 2a, left). A549 and CL1–0 cells transiently overexpressing Oct4 significantly induced *NEAT1* promoter activity (Fig. 2a, yellow bars). However, Oct4 did not affect the activity of *NEAT1* promoter with Oct4 binding element mutated in both lung cancer cells (Fig. 2a, green bars). As for the enhancer construction, reported minimal promoter activity fragment of *MALAT1* or *UCA1* [24, 25] and the enhancer fragment with Oct4 binding ChIP-seq region of *MALAT1* or *UCA1* was constructed to the upstream and downstream of luciferase reporter, respectively (Fig. 2b and c, left). A549 and CL1–0 cells transiently overexpressing Oct4 potentiated *MALAT1* and *UCA1* enhancer activities compared with activities with promoter alone (Fig. 2b and c, bars pink vs. yellow). However, the Oct4-induced enhancer activity was attenuated when the Oct4 binding element within *MALAT1* or *UCA1* enhancer was mutated in A549 and CL1–0 cells (Fig. 2b and c, bars green vs. pink). Together, we have demonstrated that Oct4 directly regulated *NEAT1*, *MALAT1* and *UCA1* lncRNAs transcription through targeting their promoter or enhancer regions.

### *NEAT1* and *MALAT1* are downstream effectors of Oct4-induced lung cancer proliferation, migration and invasion

**Fig. 3:**
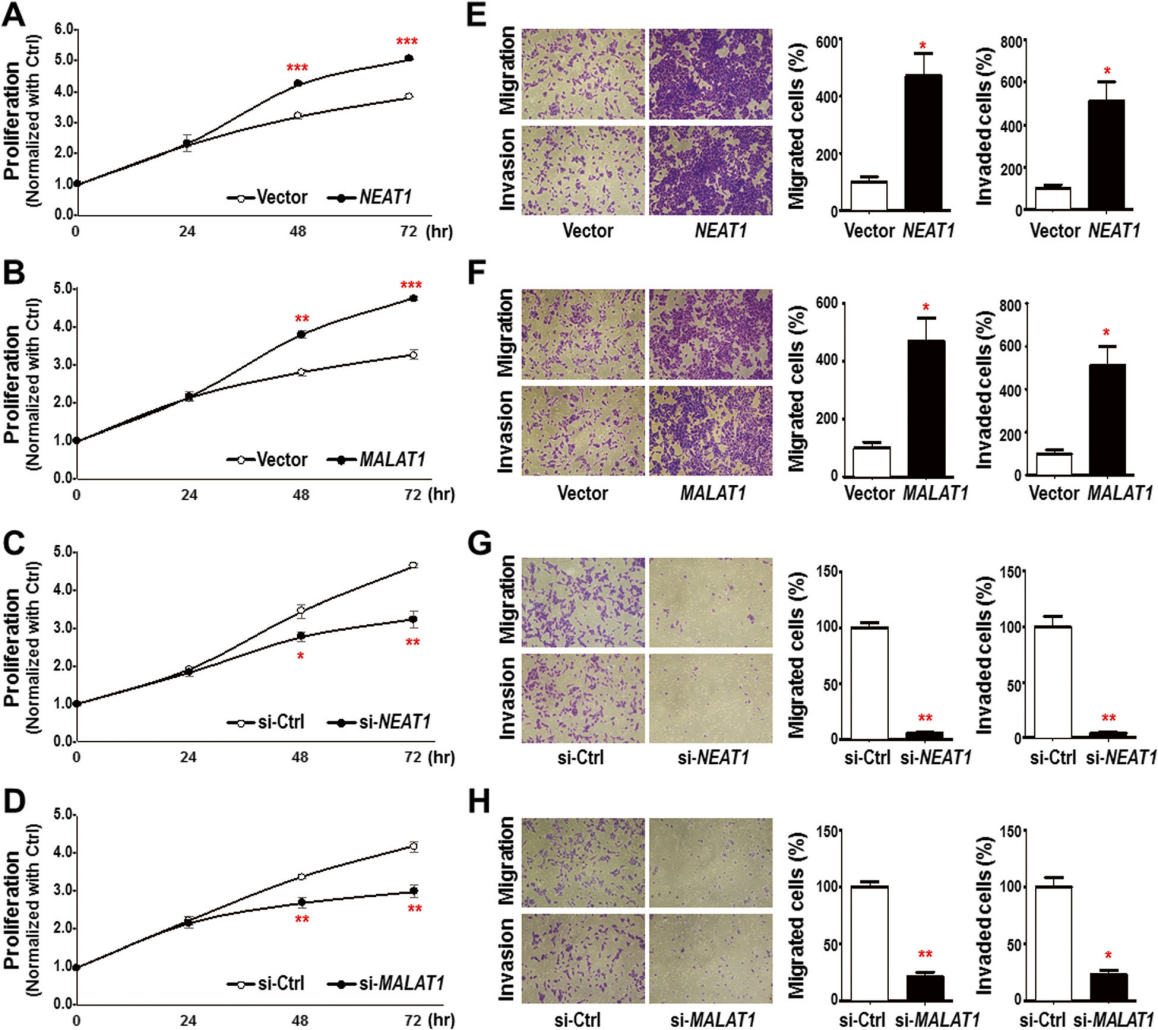
*NEAT1* and *MALAT1* promoted cell proliferation, migration and invasion in lung cancer cells. **a-d** Cell proliferation was analyzed by RealTime-Glo viability assay for A549 cells treated with *NEAT1* expression vectors (**a**), *MALAT1* expression vectors (**b**), si-*NEAT1* oligo (**c**) or si-*MALAT1* oligo (**d**) at indicated time points. **e-h** Cell motility was analyzed by transwell migration and invasion assays for A549 cells treated with *NEAT1* expression vectors (**e**), *MALAT1* expression vectors (**f**), si-*NEAT1* oligo (**g**) or si-*MALAT1* oligo (**h**). Data represent mean ± SEM. *P*-values were determined by two-tailed Student’s *t*-test. *P < 0.05; **P < 0.01; ***P < 0.001

Since oncogenic lncRNAs, *NEAT1* and *MALAT1*, were positively regulated by Oct4 at transcriptional level, we further characterized the oncogenic roles of *NEAT1* and *MALAT1* lncRNAs in lung cancer cells. Transiently overexpressing *NEAT1* or *MALAT1* in A549 cells indeed promoted cell proliferation (Fig. 3a and b). In contrast, knockdown of *NEAT1* or *MALAT1* expression suppressed cell proliferation in A549 cells (Fig. 3c and d). In addition, transwell migration and invasion assays confirmed that *NEAT1* or *MALAT1* overexpression in A549 cells enhanced cell migration and invasion (Fig. 3e and f), while knockdown of *NEAT1* or *MALAT1* suppressed cell migration and invasion (Fig. 3g and h). These results indicated that *NEAT1* and *MALAT1* indeed exerted oncogenic effects in lung cancer cells. The expression levels of *NEAT1* and *MALAT1* manipulation in functional experiments were confirmed using qRT-PCR analysis and are shown in Additional file 1: Figure S4.

**Fig. 4:**
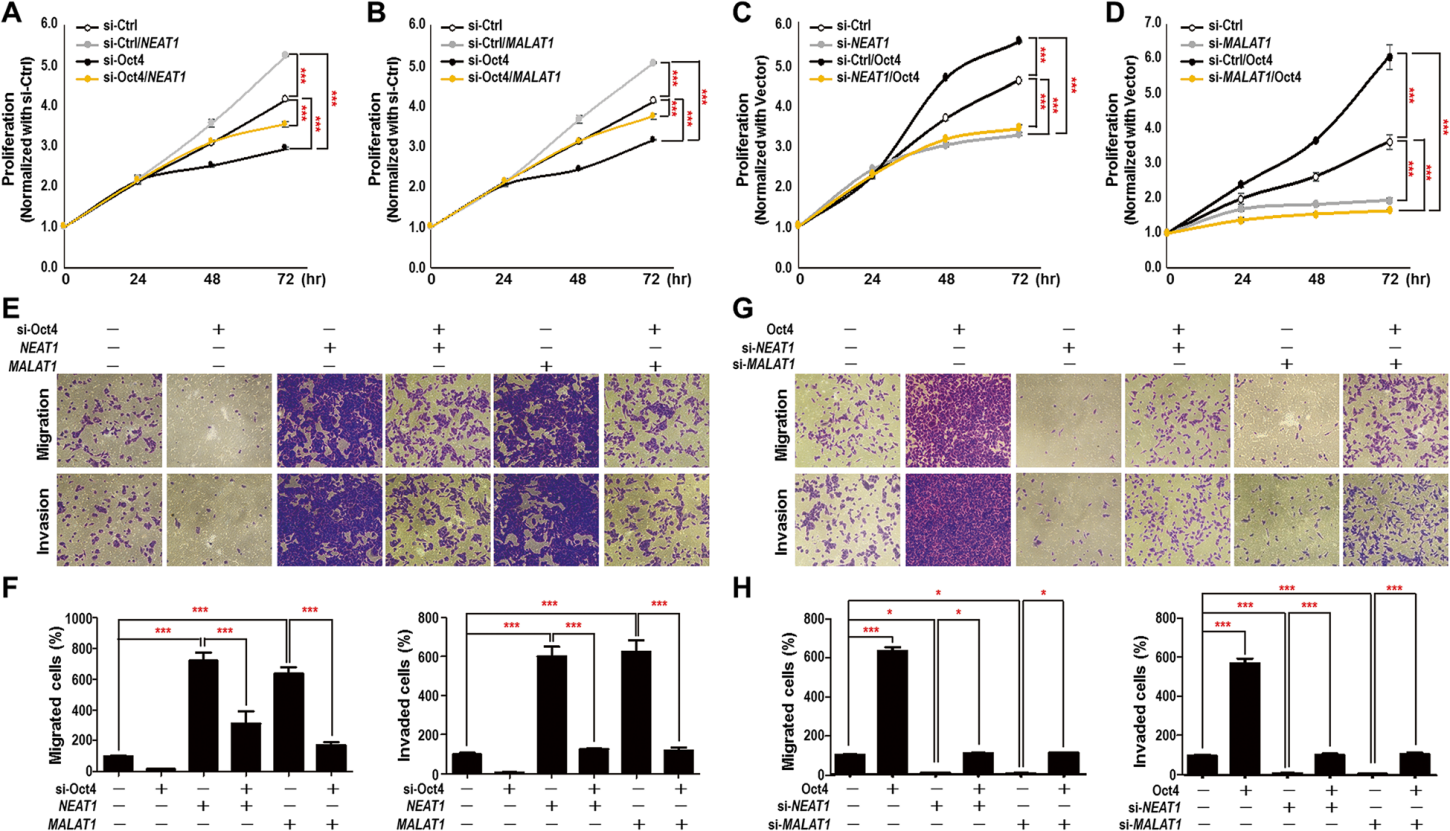
Oct4/*NEAT1* or Oct4/*MALAT1* signaling axis promoted lung cancer cell proliferation and motility. **a-d** Cell proliferation assay. Cells were reconstituted with expression vector of *NEAT1* (**a**) or *MALAT1* (**b**) in Oct4-silenced A549 cells, or si-*NEAT1* oligo (**c**) or si-*MALAT1* oligo (**d**) in Oct4-overexpressed A549 cells. Treated cells were then re-seeded and analyzed by RealTime-Glo viability assay at indicated time points. **e-h** Transwell migration and invasion assays. (**e, f**). Cells were reconstituted with expression vector of *NEAT1* or *MALAT1* in Oct4-silenced A549 cells, or (**g, h**) si-*NEAT1* oligo or si-*MALAT1* oligo in Oct4-overexpressed A549 cells. Treated cells were re-seeded and analyzed by transwell migration and invasion assays. Results were photographed and quantified. Data represent mean ± SEM. *P*-values were determined by two-way ANOVA. *P < 0.05; **P < 0.01; ***P < 0.001

To further elucidate whether *NEAT1* and *MALAT1* contributed to Oct4-mediated oncogenic effects, we conducted reconstitution experiments by transfecting A549 cells with Oct4 expression plasmid alone or together with si-*NEAT* or si-*MALAT1* oligo. On the other hand, A549 cells were transfected with si-*Oct4* oligo alone or together with *NEAT1* or *MALAT1* expression vectors. The expression level of Oct4, *NEAT1* and *MALAT1* manipulation in reconstitution experiments were confirmed using Western blot and qRT-PCR analyses and results are shown in Additional file 1: Figure S5. The transfected cells were also subjected to proliferation assay, transwell migration and invasion assays. Our results showed that reconstituted expression of *NEAT1* or *MALAT1* recovered proliferation (Fig. 4a and b), migration and invasion (Fig. 4e and f) abilities, which was downregulated in Oct4-knockdown A549 cells. In contrast, knockdown of *NEAT1* or *MALAT1* abolished Oct4-promoted A549 cells proliferation (Fig. 4c and d), migration and invasion (Fig. 4g and h) abilities. The results suggested that Oct4/*NEAT1* and Oct4/*MALAT1* transcriptional axes promote oncogenic effects in lung cancer.

### Clinical significance of coinciding high expressions of *Oct4*, *NEAT1* and *MALAT1* in lung cancer patients

To further validate Oct4/*NEAT1* and Oct4/*MALAT1* transcriptional axes in lung cancer patients, we examined RNA expression level of *Oct4*, *NEAT1* and *MALAT1* using qRT-PCR analysis of samples from 124 lung cancer patients. The overexpression rate for *Oct4*, *NEAT1* and *MALAT1* RNA were 85.5%, 90.3% and 88.7%, indicating the oncogenic roles of Oct4, *NEAT1* and *MALAT1* in lung cancer patients (Table 1). Of note, significant positive correlations were found between *Oct4* mRNA and *NEAT1* or between *Oct4* mRNA and *MALAT1* lncRNA expression (*P* < 0.001 for *Oct4* and *NEAT1*; *P* < 0.001 for *Oct4* and *MALAT1*) (Table 1).

**Table 1:** Alteration of *Oct4*, *NEAT1* and *MALAT1* expression levels in relation to clinicopathological parameters in 124 lung cancer patients

Characteristics		*Oct4*					*NEAT1*					*MALAT1*				
	Total	Normal		Overexpression			Normal		Overexpression			Normal		Overexpression		
	n	n	(%)	n	(%)	*P* ^a^	n	(%)	n	(%)	*P* ^a^	n	(%)	n	(%)	*P* ^a^
Overall	124	18	(14.5)	106	(85.5)		12	(9.7)	112	(90.3)		14	(11.3)	110	(88.7)	
Tumor stage																
I + II	72	16	(22.2)	56	(77.8)	**0.005**	11	(15.3)	61	(84.7)	**0.014**	12	(16.7)	60	(83.3)	**0.028**
III + IV	51	2	(3.9)	49	(96.1)		1	(2.0)	50	(98.0)		2	(3.9)	49	(96.1)	
T stage																
I + II	104	18	(17.3)	86	(82.7)	**0.050**	11	(10.6)	93	(89.4)	0.473	13	(12.5)	91	(87.5)	0.361
III + IV	19	0	(0.0)	19	(100.0)		1	(5.3)	18	(94.7)		1	(5.3)	18	(94.7)	
N stage^b^																
0	71	15	(21.1)	56	(78.9)	**0.021**	11	(15.5)	60	(84.5)	**0.014**	12	(16.9)	59	(83.1)	**0.029**
1 + 2	50	3	(6.0)	47	(94.0)		1	(2.0)	49	(98.0)		2	(4.0)	48	(96.0)	
M stage^c^																
0	116	18	(15.5)	98	(84.5)	0.259	12	(10.3)	104	(89.7)	0.370	14	(12.1)	102	(87.9)	0.329
1	7	0	(0.0)	7	(100.0)		0	(0.0)	7	(100.0)		0	(0.0)	7	(100.0)	
*NEAT1*																
Normal	12	10	(83.3)	2	(16.7)	**<0.001**	−	−	−	−	−	−	−	−	−	
Overexpression	112	8	(7.1)	104	(92.9)		−	−	−	−		−	−	−	−	
*MALAT1*																
Normal	14	10	(71.4)	4	(28.6)	**<0.001**	11	(78.6)	3	(21.4)	**<0.001**	−	−	−	−	
Overexpression	110	8	(7.3)	102	(92.7)		1	(0.9)	109	(99.1)		−	−	−	−	

^a^The data were analyzed by Pearson χ2 test. Bold values indicate statistical significance (*P* < 0.05)

^b^N Stage: lymph node metastasis

^c^M Stage: distant metastasis

**Fig. 5:**
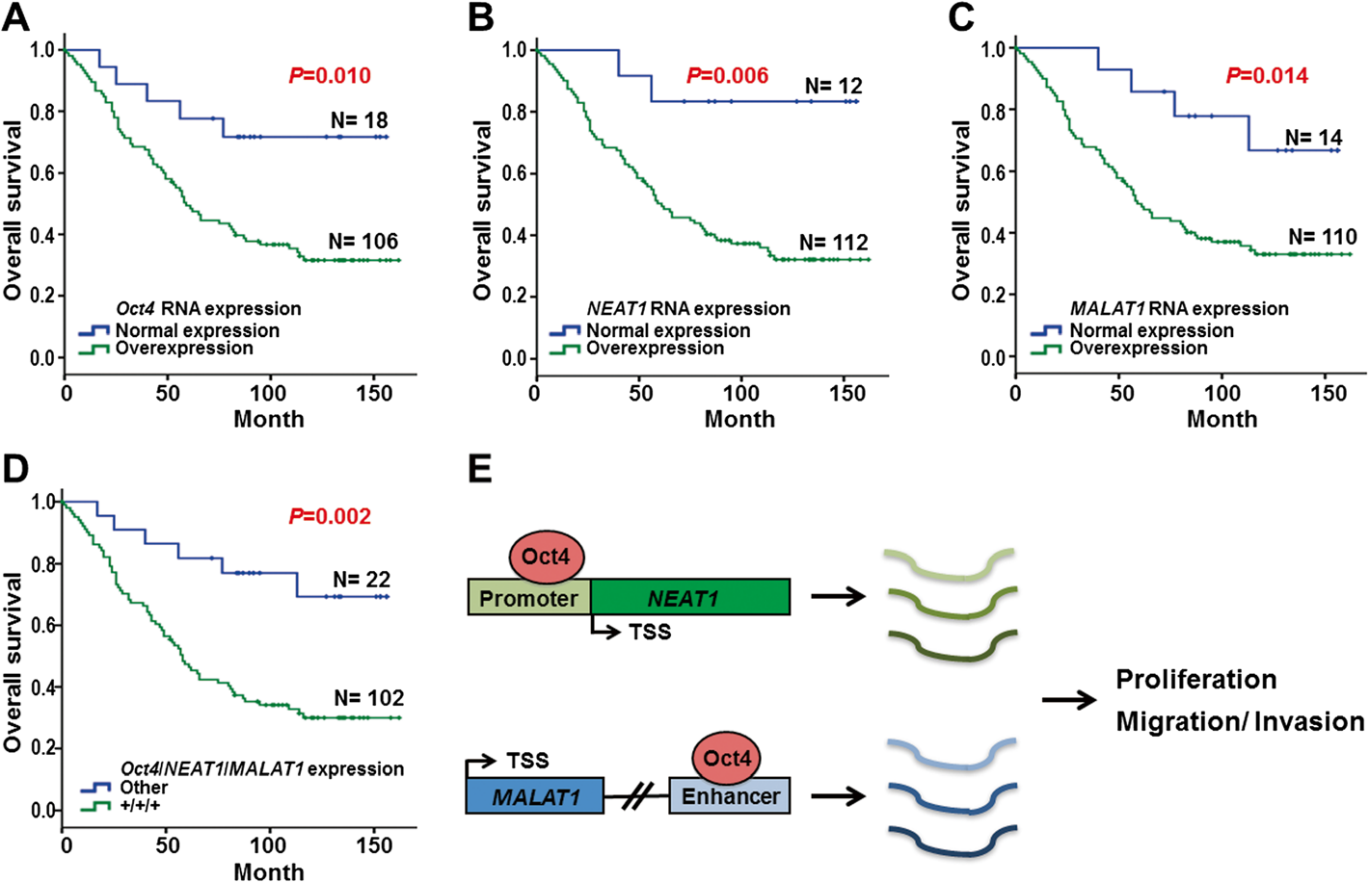
Clinical significances of Oct4/*NEAT1/MALAT1* signaling axis in 124 lung cancer patients. qRT-PCR analyses of *Oct4*, *NEAT1* and *MALAT1* RNA expression were performed in 124 lung cancer specimens. **a-d** Overall survival analyzed by Kaplan-Meier method indicated that patients with high RNA expression of *Oct4* (**a**), *NEAT1* (**b**), *MALAT1* (**c**) or coinciding high expression of *Oct4*, *NEAT1* and *MALAT1* (**d**) had poor survival. *P*-values were determined using log-rank test. **e** Schematic diagram depicting Oct4 activates *NEAT1* transcription via promoter binding and *MALAT1* transcription via enhancer binding. *NEAT1* and *MALAT1* lncRNAs act as downstream effectors of Oct4 to promote lung cancer proliferation, migration and invasion, and thereby lead to lung cancer progression

To determine whether high expression of *Oct4*, *NEAT1* and *MALAT1* contributes to poor outcome in lung cancer patients, Kaplan-Meier analysis was performed and data showed that high expression of *Oct4* mRNA (*P* = 0.01), *NEAT1* lncRNA (*P* = 0.006) or *MALAT1* lncRNA (*P* = 0.014) in lung cancer patients was associated with poor overall survival (Fig. 5a-c). Moreover, patients with coinciding high expression of *Oct4*, *NEAT1* and *MALAT1* had poorer prognosis compared with other patients (*P* = 0.002) (Fig. 5d). Univariate Cox regression analysis confirmed that patients with co-overexpressed *Oct4*, *NEAT1* and *MALAT1* had poor outcome (*P* = 0.016, hazard ratio = 2.78, 95% confidence interval = 1.21–6.42; Table 2, left panel). After adjusting for late stage and lymph node metastasis using multivariate Cox regression analysis, co-overexpression of *Oct4*, *NEAT1* and *MALAT1* in lung cancer patients showed a relative risk of death of 2.42 (*P* = 0.039; Table 2, right panel). These clinical studies clearly indicated that Oct4 positively correlates with *NEAT1* and *MALAT1* expression in lung cancer and that Oct4/*NEAT1*/*MALAT1* co-overexpression is an independent factor for prediction of poor outcome in lung cancer patients.

**Table 2:** Cox regression analysis of risk factors for cancer-related death in lung cancer patients

Characteristics	Univariate analysis		Multivariate analysis	
	HR^a^ (95% CI^b^)	*P*-value^c^	HR^a^ (95% CI^b^)	*P*-value^c^
*Oct4*/*NEAT1*/*MALAT1* expression				
Other	1.00		1.00	
+/+/+	2.78 (1.21–6.42)	**0.016**	2.42 (1.05–5.62)	**0.039**
Age				
< 65 year-old	1.00		- ^e^	
≥ 65 year-old	1.03 (0.64–1.67)	0.896	- ^e^	- ^e^
Gender				
Female	1.00		- ^e^	
Male	1.50 (0.86–2.61)	0.150	- ^e^	- ^e^
Smoking habit				
Non-smoker	1.00		- ^e^	
Smoker	0.96 (0.54–1.72)	0.891	- ^e^	- ^e^
Tumor type^d^				
SCC	1.00		- ^e^	
ADC	1.32 (0.74–2.37)	0.343	- ^e^	- ^e^
Stage				
Stage I-II	1.00		1.00	
Stage III-IV	1.86 (1.18–2.95)	**0.008**	1.13 (0.65–1.97)	0.672
T stage				
T1–2	1.00		- ^e^	
T3–4	1.42 (0.78–2.59)	0.249	- ^e^	- ^e^
N stage				
N0	1.00		1.00	
≥ N1	2.36 (1.48–3.78)	**<0.001**	1.93 (1.10–3.38)	**0.022**
M stage				
M0	1.00		- ^e^	
≥ M1	1.98 (0.86–4.57)	0.110	- ^e^	- ^e^

^a^ *HR* hazard ratio

^b^ *CI* confidence interval

^c^Bold values indicate statistical significance (*P* < 0.05)

^d^ *SCC* squamous cell carcinoma, *ADC* adenocarcinoma

^e^The variables without significant HR in the univariate analysis were not included in the multivariate analysis

## Discussion

In the present study, we have revealed that Oct4 binds to the genomic loci of lncRNAs through ChIP-seq and bioinformatic analysis (Fig. 1). We then validated that Oct4 bound on the promoter or enhancer regions of lncRNAs (Fig. 1). Dual luciferase activity assay further confirmed that Oct4 potentiated promoter activity of *NEAT1* and enhancer activities of *MALAT1* and *UCA1* lncRNAs (Fig. 2). Moreover, *NEAT1* and *MALAT1* acted as downstream effectors of Oct4 to promote proliferation, migration and invasion abilities of A549 lung cancer cells (Figs. 3 and 4). Of note, positive correlations between *Oct4* mRNA and *NEAT1*/*MALAT1* lncRNAs were evident in lung cancer patient specimens (Fig. 5). Our study provides new evidence that Oct4 transcriptionally regulates lncRNAs expression by targeting their promoter or enhancer regions. *NEAT1* and *MALAT1* function as Oct4 downstream mediators to promote lung cancer proliferation, migration and invasion (Fig. 5e).

Recently, the roles of *NEAT1* in cancer have been uncovered. Studies have reported that *NEAT1* is upregulated in prostate cancer, colorectal cancer and lung cancer, and thus associated with poor prognosis in these cancer patients [16, 26–28]. Rubin and associates demonstrated that estrogen receptor transcriptionally activates *NEAT1* expression to promote prostate tumorigenesis under the treatment of oestrogen [16]. *NEAT1* has also been shown to modulate prostate cancer-specific gene expression through chromatin modifications and thus contributes to cancer progression [16]. However, the upstream mechanisms of *NEAT1* overexpression in cancers await to be uncovered. It is until recently that HIF-2α is demonstrated to transactivate *NEAT1* transcription under hypoxia, which promotes the formation of paraspeckles, accelerates tumor proliferation and cancer cell survival leading to poor prognosis in breast cancer patient [29]. In our study, we have shown that a well-known stemness transcription factor Oct4 transcriptionally upregulates *NEAT1* expression through binding to Oct4 consensus binding element on promoter region (Figs. 1 and 2a), and therefore promoting lung cancer proliferation and motility (Figs. 3 and 4). Notably, *NEAT1* is found overexpressed in BRCA1-deficient breast cancer and promotes self-renewal abilities in breast cancer cells through epigenetically suppressing miR-129-5p, which targets to Wnt4 [30]. The last-mentioned study together with our results revealed a potential role of *NEAT1* in maintaining stemness properties and suggested that Oct4-mediated *NEAT1* upregulation may play critical roles in embryonic or cancer stemness maintenance.

*MALAT1* was first identified as a prognosis marker in early-stage metastasizing lung cancer [31]. *MALAT1* knockdown in lung cancer cells decreases cell migration abilities [32]. In addition, *MALAT1* suppresses expression of anti-metastasis genes such as *MIA2* (melanoma inhibitory activity 2) and *ROBO1* (roundabout 1), while induces pro-metastasis genes including *LPHN2* (latrophilin 2) and *ABCA1* (ATP-binding cassette, sub-family A, member 1) to accelerate metastasis [33]. However, *MALAT1*-promoting lung cancer cell proliferation in different studies are contradictory. For example, *MALAT1* has no effect on cell proliferation in vitro and slightly promotes tumor growth in vivo [33]. In contrast, knockdown of *MALAT1* in A549 lung cancer cells decreased proliferation [34], which is consistent with our results that *MALAT1* plays a role in lung cancer cell proliferation (Fig. 3b and d) and this provides new insight into the role of *MALAT1* in various cancer types. *MALAT1* has been demonstrated to promote lung, bladder, colorectal, liver, oral and prostate cancer cells proliferation and migration [32, 33, 35–40]. However, the upstream regulatory mechanisms of *MALAT1* expression remain unclear, especially at the transcription level. Recently, Sp1 is found to transcriptionally activate *MALAT1* expression through targeting the promoter region and the Sp1-*MALAT1* axis may play a critical role in cancers [34]. In addition, Wnt signaling pathway acts upstream of *MALAT1* transcription, which is mediated by TCF4 binding on *MALAT1* promoter in endometrioid endometrial cancer [41]. Importantly, our results provide the first evidence of Oct4-mediated *MALAT1* upregulation through enhancer regions.

## Conclusions

In conclusion, our genome-wide ChIP-seq analysis reveals a novel role of Oct4 transcription regulation on lncRNAs in lung cancer. We demonstrate for the first time that Oct4 promotes transcription of *NEAT1*, *MALAT1* and *UCA1* through targeting their promoter or enhancer regions. Moreover, *NEAT1* and *MALAT1* function as Oct4 downstream mediators to promote lung cancer proliferation, migration and invasion. Clinical studies confirm that patients with Oct4/*NEAT1*/*MALAT1* high expression had poor outcome. Collectively, our study provides a novel insight into Oc4-lncRNAs as critical axes in lung tumorigenesis.

## Additional file

Additional file 1: Table S1. Supplementary methods. Anchorage-independent growth assay, Tumor-sphere formation assay, Tumor formation assay, Western blot analysis. **Table S1.** The plasmids and their characteristics used in the current study. **Table S2.** The ChIP-PCR primers used in the current study. **Table S3.** The cDNA primers used in the current study. **Table S4.** The construction primers of promoters and enhancers used in the current study. **Figure S1.** Oct4 promoted lung cancer tumorigenesis in vitro and in vivo*.* **A** Anchorage-independent assays in empty vector stably-transfected cell line (vector) and two biological replicates of Oct4 stably-overexpressed A549 and CL1-0 cell lines (Oct4#1, Oct4#2). Results were photographed (left) and quantified (right). **B** Transwell migration and invasion assay analysis of stably-transfected cell lines in A549 and CL1-0 cells. Results were photographed (left) and quantified (right). *P*-values were determined by two-tailed Student’s *t*-test. **P* < 0.05; ***P* < 0.01; ****P* < 0.001. **C** In vitro tumor sphere formation assay of A549 lung cancer cells stably expressing Oct4#1 or vector photographed (top) and quantified (middle). In vivo tumor formation assay using limited cell number (100, 1000, and 5000 cells) of vector and Oct4#1 cells. Tumor incidence of mice was analyzed at 8 weeks after implantation. **D** The immunoblots (upper) and qRT-PCR (lower) confirmed Oct4 expression in A549 and CL1–0 stable clones. **Figure S2.** Expression of lncRNAs in CL1–0 lung cancer cells manipulated for Oct4*.* **A**, **B** qRT-PCR analysis of eight lncRNAs expression in CL1-0 cells stably overexpressing Oct4 (Oct4#1, Oct4#2) (**A**) or Oct4-silenced CL1-0 cells (si-Oct4#1, si-Oct4#2) (**B**). Target lncRNA expression levels were normalized to *GAPDH* expression levels. Data represent mean ± SEM. *P*-values were determined by two-tailed Student’s *t*-test. **P* < 0.05; ***P* < 0.01; ****P* < 0.001. **Figure S3.** Oct4 positively regulated *NEAT1* and *UCA1* lncRNAs transcription in normal bronchial epithelial BEAS-2B cells*.* qRT-PCR analysis of selected lncRNAs expressions in BEAS-2B cells overexpressing Oct4. Target lncRNA expression levels were normalized to *GAPDH* expression levels. Data represent mean ± SEM. *P*-values were determined by two-tailed Student’s *t*-test. **P* < 0.05; ***P* < 0.01; ****P* < 0.001. **Figure S4.** RNA expression level of the manipulated *NEAT1* and *MALAT1* in A549 lung cancer cells. A549 cells transfected with *NEAT1* expression vector (**A**) or *MALAT1* expression vector (**B**), si-*NEAT1* oligo (si-*NEAT1*) (**C**) or si-*MALAT1* oligo (si-*MALAT1*) (**D**) were harvested and subjected to qRT-PCR assays for *NEAT1* and *MALAT1* RNA expression. Data are mean ± SEM. *P*-values were determined by two-tailed Student’s *t*-test. **P* < 0.05; ****P* < 0.001. **Figure S5.** RNA and protein expression level of the manipulated Oct4, *NEAT1* and *MALAT1* in A549 lung cancer cells. A549 cells were transfected with expression vectors of *NEAT1* (**A**) or *MALAT1* (**B**) alone or together with si-Oct4 oligo (si-Oct4). A549 cells were transfected with si-*NEAT1* oligo (si-*NEAT1*) (**C**) or si-*MALAT1* oligo (si-*MALAT1*) (**D**) alone or together with Oct4 expression vector. Cell lysates were subjected to qRT-PCR assays for *Oct4*, *NEAT1* and *MALAT1* RNA expression or Western blot analysis for Oct4 protein expression (inset). GAPDH serves as an internal control. Data are mean ± SEM. *P*-values were determined by two-way ANOVA. ***P* < 0.01; ****P* < 0.001. (PDF 911 kb)

## Abbreviations

ChIP: Chromatin-immunoprecipitation
lncRNA: Long non-coding RNA
*MALAT1*: *Metastasis-associated lung adenocarcinoma transcript 1*
*NEAT1*: *Nuclear paraspeckle assembly transcript 1*
*POU5F1*: POU domain, class 5, transcription factor 1
*UCA1*: *Urothelial carcinoma-associated 1*
