# Supplementary material for: Oct4 transcriptionally regulates the expression of long non-coding RNAs NEAT1 and MALAT1 to promote lung cancer progression
Source: Mol Cancer. 2017 Jun 14;16:104. doi: 10.1186/s12943-017-0674-z (PMC5471675; doi:10.1186/s12943-017-0674-z)
Supplement: Additional file 1: Table S1. — Supplementary methods. Anchorage-independent growth assay, Tumor-sphere formation assay, Tumor formation assay, Western blot analysis. Table S1. The plasmids and their characteristics used in the current study. Table S2. The ChIP-PCR primers used in the current study. Table S3. The cDNA primers used in the current study. Table S4. The construction primers of promoters and enhancers used in the current study. Figure S1. Oct4 promoted lung cancer tumorigenesis in vitro and in vivo. A Anchorage-independent assays in empty vector stably-transfected cell line (vector) and two biological replicates of Oct4 stably-overexpressed A549 and CL1-0 cell lines (Oct4#1, Oct4#2). Results were photographed (left) and quantified (right). B Transwell migration and invasion assay analysis of stably-transfected cell lines in A549 and CL1-0 cells. Results were photographed (left) and quantified (right). P-values were determined by two-tailed Student’s t-test. *P < 0.05; **P < 0.01; ***P < 0.001. C In vitro tumor sphere formation assay of A549 lung cancer cells stably expressing Oct4#1 or vector photographed (top) and quantified (middle). In vivo tumor formation assay using limited cell number (100, 1000, and 5000 cells) of vector and Oct4#1 cells. Tumor incidence of mice was analyzed at 8 weeks after implantation. D The immunoblots (upper) and qRT-PCR (lower) confirmed Oct4 expression in A549 and CL1–0 stable clones. Figure S2. Expression of lncRNAs in CL1–0 lung cancer cells manipulated for Oct4. A, B qRT-PCR analysis of eight lncRNAs expression in CL1-0 cells stably overexpressing Oct4 (Oct4#1, Oct4#2) (A) or Oct4-silenced CL1-0 cells (si-Oct4#1, si-Oct4#2) (B). Target lncRNA expression levels were normalized to GAPDH expression levels. Data represent mean ± SEM. P-values were determined by two-tailed Student’s t-test. *P < 0.05; **P < 0.01; ***P < 0.001. Figure S3. Oct4 positively regulated NEAT1 and UCA1 lncRNAs transcription in normal bronchial epithelial BEAS-2B cells. qRT-PCR anal [file 12943_2017_674_MOESM1_ESM.pdf]

Additional file 1

Oct4 transcriptionally regulates the expression of long non-coding RNAs *NEAT1* and *MALAT1* to promote lung cancer progression

Jayu Jen[1,2], Yen-An Tang[1,2], Ying-Hung Lu[1], Che-Chung Lin[1], Wu-Wei Lai[3] and Yi-Ching Wang[1,2]*

[1]Department of Pharmacology, College of Medicine, National Cheng Kung University, Tainan 701, Taiwan

[2]Institute of Basic Medical Sciences, College of Medicine, National Cheng Kung University, Tainan 701, Taiwan

[3]Division of Thoracic Surgery, Department of Surgery, National Cheng Kung University Hospital, College of Medicine, National Cheng Kung University, Tainan 701, Taiwan

Supplementary methods

Anchorage-independent growth assay

Anchorage-independent growth assay was performed by seeding A549 and CL1-0 at 3×10^3 cells/well in 0.4% bactoagar on a bottom layer of solidified 0.6% bactoagar in 6-well plates. After 12~17 days for A549 cells and 7~10 days for CL1-0 cells, colonies consisted of > 30 cells were counted.

Tumor-sphere formation assay

Cells were expanded as spheres in a 10-cm ultra-low adhesion culture dish (Corning Inc., Corning, NY, USA) containing DMEM/F-12 with N2 supplement (Invitrogen), 20 ng/ml epithelial growth factor (EGF), and 20 ng/ml basic fibroblast growth factor (bFGF; PeproTech Inc., Rocky Hill, NJ, USA), referred to as stem cell medium. Tumor spheres consisting of > 30 cells were counted and expressed as the means ± SEM of triplicate within the same experiment.

Tumor formation assay

5-6-week-old BALB/c nude female mice were subcutaneously implanted with varying number (1×10^2, 1×10^3, or 5×10^3) of vector control (vector) or Oct4-stably expressing A549 cells (Oct4#1). Vector and Oct4#1 cells in 50 μl Hanks' balanced salt solution (HBSS) were mixed with 50 μl matrigel (2.5 mg/ml, Sigma-Aldrich, St. Louis, MO, USA), and then subcutaneously injected into the flanks of mice. The incidence of tumor formation was monitored within 8 weeks after implantation.

Western blot analysis

Samples containing equal amounts of protein (50 μg) were separated on an 8% sodium dodecylsulphate-polyacrylamide gel electrophoresis (SDS-PAGE) and electroblotted onto Immobilon-P membranes (Millipore Co., Billerica, MA, USA). Immunoblotting was performed by incubating membranes with a 1:1000 dilution of anti-Oct4 antibody (Abcam, Cambridge, UK; Cat. ab-19857) overnight. GAPDH expression was used as internal control. Proteins expression was detected with indicated secondary antibody for 1 h at room temperature.

Supplementary tables

Table S1. The plasmids and their characteristics used in the current study.

Plasmid	Target	Insert (bp)	Function	Source
pPyCAGIP-vector	--[c]	0	Vector control	From Dr. Ying Jin[d]
pPyCAGIP-Oct4	Wild-type Oct4	1,083	Overexpression	From Dr. Ying Jin[d]
pMIR-REPORT-vector	--[c]	0	Vector control	From Dr. Tetsuro Hirose[e]
pMIR-REOPRT-NEAT1	*NEAT1*	3,756	Overexpression	From Dr. Tetsuro Hirose[e]
pCMV-vector	--[c]	0	Vector control	From Dr. Kannanganattu V. Prasanth[f]
pCMV-MALAT1	*MALAT1*	7,161	Overexpression	From Dr. Kannanganattu V. Prasanth[f]
pGL3-Basic vector	--[c]	0	Vector control	Promega
pGL3-NEAT1 WT[a]	*NEAT1* promoter (WT)	4,422	Promoter activity assay	Homemade
pGL3-NEAT1 Mut[b]	*NEAT1* promoter (Mut)	4,422	Promoter activity assay	Homemade
pGL3-MALAT1-pro	*MALAT1* promoter-luc	468	Enhancer activity assay	Homemade
pGL3-MALAT1-enh WT[a]	*MALAT* promoter-luc-enhancer (WT)	435	Enhancer activity assay	Homemade
pGL3-MALAT1-enh Mut[b]	*MALAT* promoter-luc-enhancer (Mut)	435	Enhancer activity assay	Homemade
pGL3-UCA1-pro	*UCA1* promoter-luc	519	Enhancer activity assay	Homemade
pGL3-UCA1-enh WT[a]	*UCA1* promoter-luc-enhancer (WT)	512	Enhancer activity assay	Homemade
pGL3-UCA1-enh Mut[b]	*UCA1* promoter-luc-enhancer (Mut)	512	Enhancer activity assay	Homemade

[a] WT: wild-type Oct4 binding sites within *NEAT1* promoter, *MALAT1* enhancer and *UCA1* enhancer

[b] Mut: mutated Oct4 binding sites within *NEAT1* promoter, *MALAT1* enhancer and *UCA1* enhancer

[c] Not applicable.

[d] Plasmid was kindly provided by Dr. Ying Jin at Shanghai Stem Cell Institute, Shanghai Jiao Tong University School of Medicine, Shanghai, China.

[e] Plasmid was kindly provided by Dr. Tetsuro Hirose at Institute for Genetic Medicine, Hokkaido University, Sapporo, Japan.

[f] Plasmid was kindly provided by Dr. Kannanganattu V. Prasanth at Department of Cell and Developmental Biology, University of Illinois at Urbana-Champaign, Urbana, USA.

Table S2. The ChIP-PCR primers used in the current study.

Gene	Primer	Sequences (5' → 3')	Application[a]	PCR size (bp)	Tm (°C)
BACE1AS	Forward	TGG TGG GTG TTG ACT GTG ACA	ChIP-PCR	150	54
	Reverse	AAA CTC ACC TGC CGA CAA GCT			
IGF2AS	Forward	CTC AAT TGT TTT GTG AAG GGA AAA	ChIP-PCR	157	50
	Reverse	AAT GTA CAT ATG GAC CCC CAA GTC			
MALAT1	Forward	CCT GGC CAC AGT TGC GTA T	ChIP-PCR	150	61
	Reverse	GTG AAC CGA GAT CGC AAC ACT			
NEAT1	Forward	AGG ACA GTT GGG AAG GAG GAA	ChIP-PCR	188	65
	Reverse	GTG CAC TCT CAG CCA CAC GTT			
SRA	Forward	GGG CAC AGT CCT GGA ATC AG	ChIP-PCR	150	61
	Reverse	AGT AGG GCA AGC GTT TTT ATC CT			
TUG1	Forward	ACC ACT TTG GCA GAG CCT TTT	ChIP-PCR	200	65
	Reverse	TGA ATG GGA ACA AGC ACA CAA			
UCA1	Forward	TGC TTC TGC TTC ATC CCA GTA G	ChIP-PCR	202	61
	Reverse	CCT GTA GCG GAG ACA GAG ACA GT			
GAS5	Forward	AAT GAT ATG TAA GAA AAG GAG GCT ATA GG	ChIP-PCR	123	54
	Reverse	GAG GCT TGG GAA ATA GTG GTA GTA GA			

[a] ChIP-PCR: Chromatin immunoprecipitation coupled with polymerase chain reaction.

Table S3. The cDNA primers used in the current study.

Gene	Primer	Sequences (5’→ 3’)	Application[a]	PCR size (bp)	Tm (°C)
BACE1AS	Forward	GAA GGG TCT AAG TGC AGA CAT CTT	qRT-PCR	61	60
	Reverse	AGG GAG GCG GTG AGA GT			
GAPDH	Forward	GAG TCA ACG GAT TTG GTC GT	qRT-PCR	238	60
	Reverse	TTG ATT TTG GAG GGA TCT CG			
IGF2AS	Forward	AGC TCT GCT TGA CGA GGC CA	qRT-PCR	119	60
	Reverse	TCT GTT GCA CCC TGG ACC CA			
MALAT1	Forward	TCTCCCCACAAGCAACTTCT	qRT-PCR	150	60
	Reverse	ACCTCGACACCATCGTTACC			
NEAT1	Forward	TCG GGT ATG CTG TTG TGA AA	qRT-PCR	95	60
	Reverse	TGA CGT AAC AGA ATT AGT TCT TAC CA			
Oct4	Forward	CGA AAG AGA AAG CGA ACC AG	qRT-PCR	157	60
	Reverse	GCC GGT TAC AGA ACC ACA CT			
SRA	Forward	AGG ATG GAT CCC CCA GAG T	qRT-PCR	87	60
	Reverse	TGG GAG CCT TAC TTG AAG GAG			
TUG1	Forward	TAG CAG TTC CCC AAT CCT TG	qRT-PCR	116	60
	Reverse	CAC AAA TTC CCA TCA TTC CC			
UCA1	Forward	CTT CTG CAT AGG ATC TGC AAT CAG	qRT-PCR	136	60
	Reverse	TTT TGT CCC CAT TTT CCA TCA TAC G			
GAS5	Forward	AGC TGG AAG TTG AAA TGG	qRT-PCR	149	60
	Reverse	CAA GCC GAC TCT CCA TAC C			

[a] qRT-PCR: Quantitative reverse-transcriptase polymerase chain reaction

Table S4. The construction primers of promoters and enhancers used in the current study.

Gene[a]	Primer	Sequences (5' → 3')	Application[b]	PCR size (bp)	Tm (°C)
NEAT1 promoter WT	Forward	AAG GTA CCA GGA CAG TTG GGA AGG AGG AAG	Promoter construct PCR	4,422	61
	Reverse	AGC TAG CCT TCC TCC CCC ACA ACT ACA CC			
NEAT1 promoter-1[c] Mut	Forward	AAG GTA CCA GGA CAG TTG GGA AGG AGG AAG GGC CCG T	Site-directed mutagenesis PCR	118	54
	Reverse	GCC CTG GTT TAC GGA TCC GAG GTG AGG TGA			
NEAT1 promoter-2 Mut	Forward	TCA CCT CAC CTC GGA TCC GTA AAC CAG GGC	Site-directed mutagenesis PCR	4,334	54
	Reverse	AGC TAG CCT TCC TCC CCC ACA ACT ACA CCC AGG CGC			
MALAT1 promoter	Forward	AAG GTA CCA GAG CCG GTT AGA ACC AGT G	Promoter construct PCR	468	61
	Reverse	CTA AGC TTT CCT CCA AAC CCC			
*MALAT*1 enhancer WT	Forward	TGG ATC CGC CTT CCA AAG TGC TGA GAT	Enhancer construct PCR	435	54
	Reverse	CCC GTC GAC CCA ACA ACA ATG GCA AGA AA			
*MALAT*1 enhancer Mut	Forward	TGA AGT GTC TGT TCA TCC CGG TTG TGT TTT TCT CTT	Site-directed mutagenesis PCR	5,721	54
	Reverse	AAG AGA AAA ACA CAA CCG GGA TGA ACA GAC ACT TCA			
UCA1 promoter	Forward	AAG GTA CCA GAA ATG ACC CAG GAG CTG A	Promoter construct PCR	519	65
	Reverse	CTA AGC TTT CAG CGA AGG GAG ATA GGA G			
UCA1 enhancer WT	Forward	TGG ATC CTC ATC CCA GTA GGA GGC TCT	Enhancer construct PCR	512	65
	Reverse	CCC GTC GAC ACG TGT GTG TGT GTT GGT TTT			
UCA1 enhancer Mut	Forward	ACT GAG CCC AAA TCG CCT TAT TTA TCC CTC	Site-directed mutagenesis PCR	5,849	50
	Reverse	GAG GGA TAA ATA AGG CGA TTT GGG CTC AGT			

[a] WT: Wild-type Oct4 binding site; Mut: Mutant Oct4 binding site

[b] PCR: polymerase chain reaction

[c] The PCR fragments promoter-1 and promoter-2 were ligated and then used to construct *NEAT1* promoter mutant vector.

Supplementary figure and figure legend

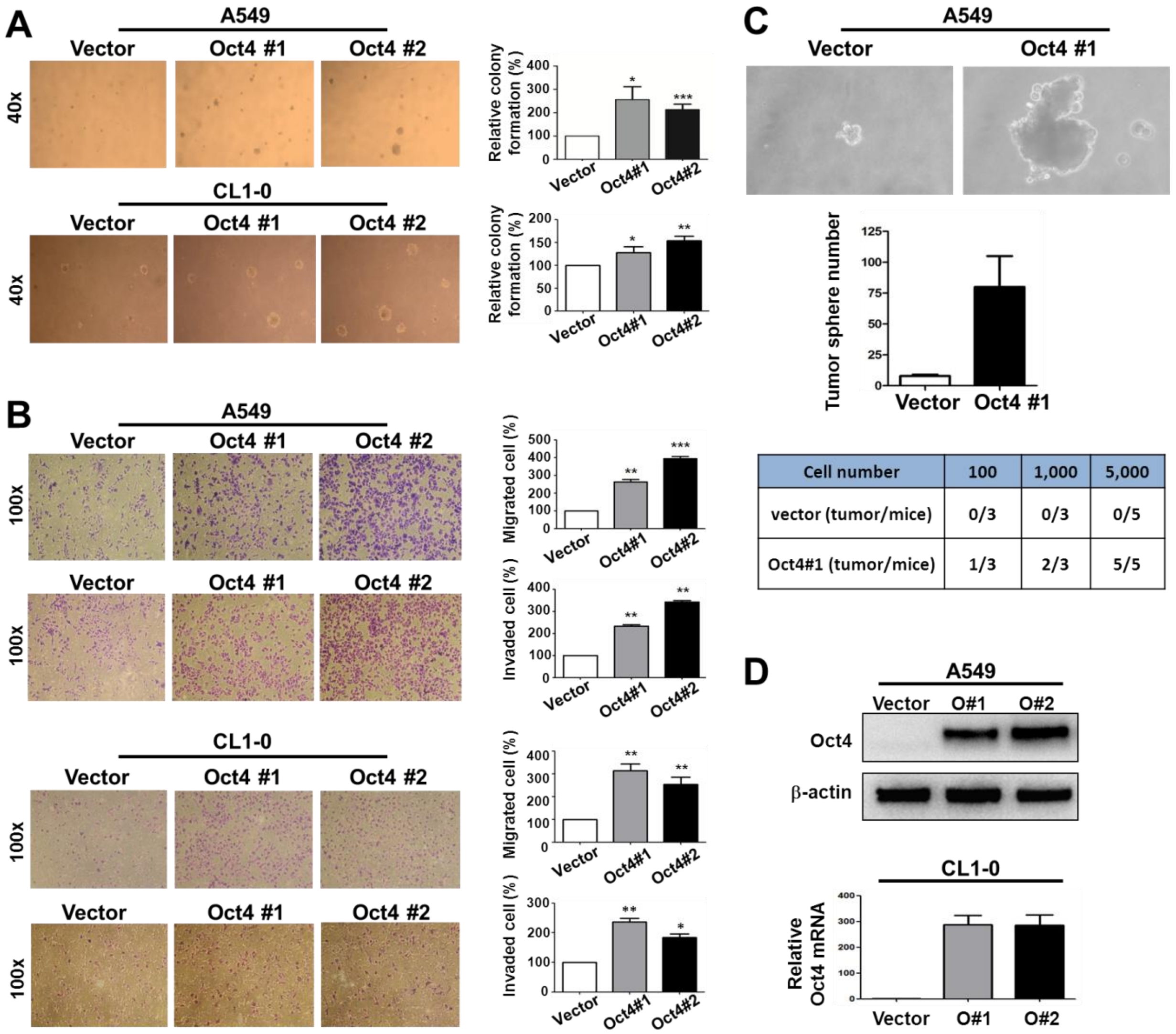

Cell number	100	1,000	5,000
vector (tumor/mice)	0/3	0/3	0/5
Oct4#1 (tumor/mice)	1/3	2/3	5/5

Figure S1 Oct4 promoted lung cancer tumorigenesis *in vitro* and *in vivo.* **A** Anchorage-independent assays in empty vector stably-transfected cell line (vector) and two biological replicates of Oct4 stably-overexpressed A549 and CL1-0 cell lines (Oct4#1, Oct4#2). Results were photographed (left) and quantified (right). **B** Transwell migration and invasion assay analysis of stably-transfected cell lines in A549 and CL1-0 cells. Results were photographed (left) and quantified (right). *P*-values were determined by two-tailed Student's *t*-test. *$P < 0.05$; **$P < 0.01$; ***$P < 0.001$. **C** *In vitro* tumor sphere formation assay of A549 lung cancer cells stably expressing Oct4#1 or vector photographed (top) and quantified (middle). *In vivo* tumor formation assay using limited cell number (100, 1000, and 5000 cells) of vector and Oct4#1 cells. Tumor incidence of mice was analyzed at 8 weeks after implantation. **D** The immunoblots (upper) and qRT-PCR (lower) confirmed Oct4 expression in A549 and CL1-0 stable clones.

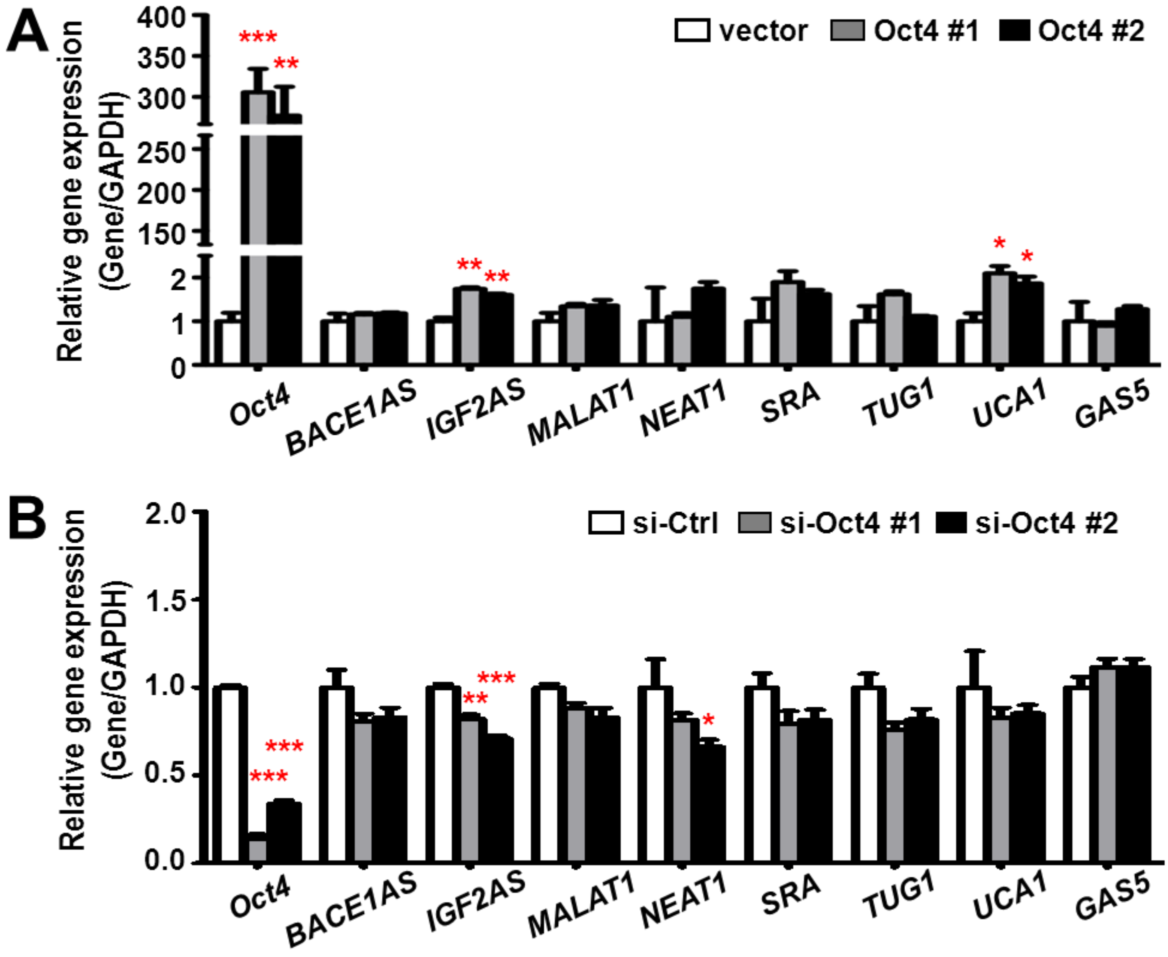

Figure S2 Expression of lncRNAs in CL1-0 lung cancer cells manipulated for Oct4. **A**, **B** qRT-PCR analysis of eight lncRNAs expression in CL1-0 cells stably overexpressing Oct4 (Oct4#1, Oct4#2) (**A**) or Oct4-silenced CL1-0 cells (si-Oct4#1, si-Oct4#2) (**B**). Target lncRNA expression levels were normalized to *GAPDH* expression levels. Data represent mean ± SEM. *P*-values were determined by two-tailed Student's *t*-test. **P* < 0.05; ***P* < 0.01; ****P* < 0.001.

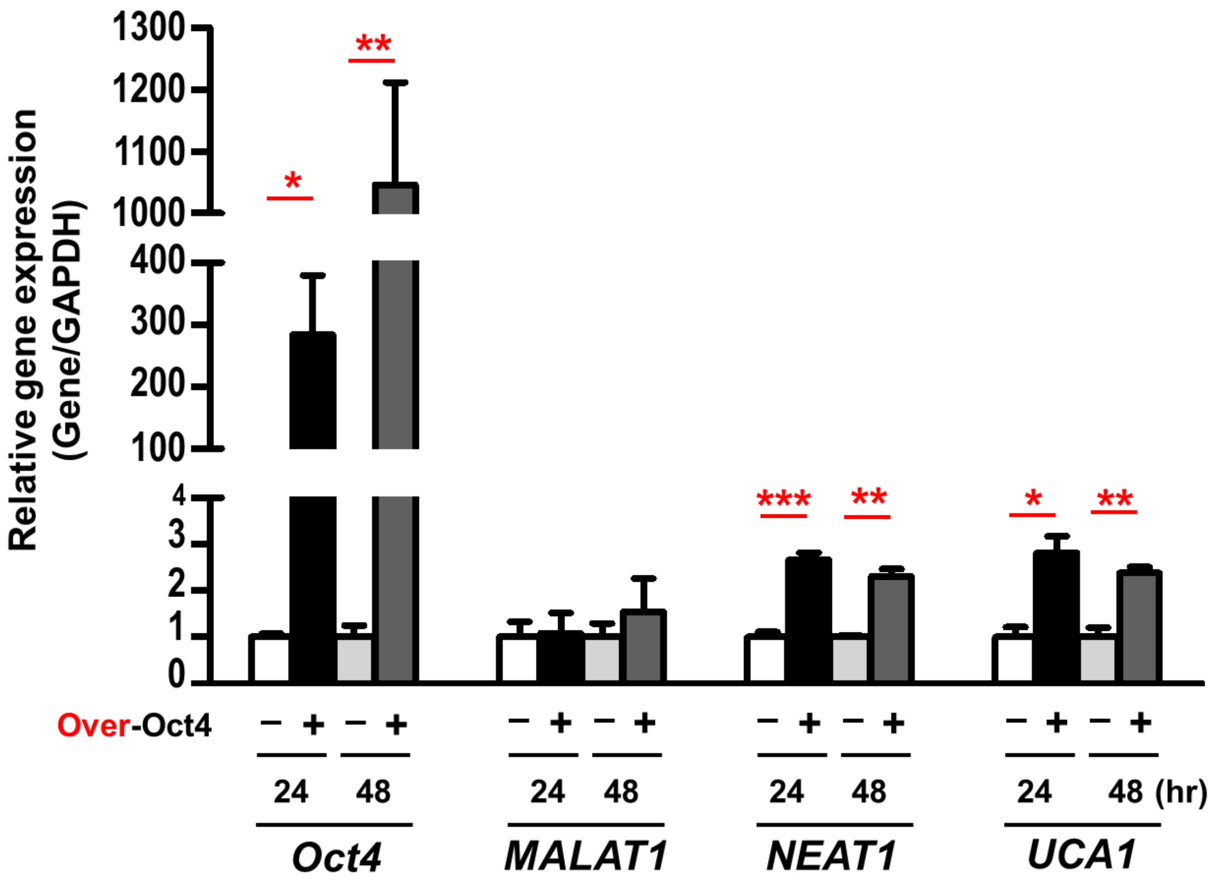

Figure S3 Oct4 positively regulated *NEAT1* and *UCA1* lncRNAs transcription in normal bronchial epithelial BEAS-2B cells. qRT-PCR analysis of selected lncRNAs expressions in BEAS-2B cells overexpressing Oct4. Target lncRNA expression levels were normalized to *GAPDH* expression levels. Data represent mean ± SEM. *P*-values were determined by two-tailed Student's *t*-test. *$P < 0.05$; **$P < 0.01$; ***$P < 0.001$.

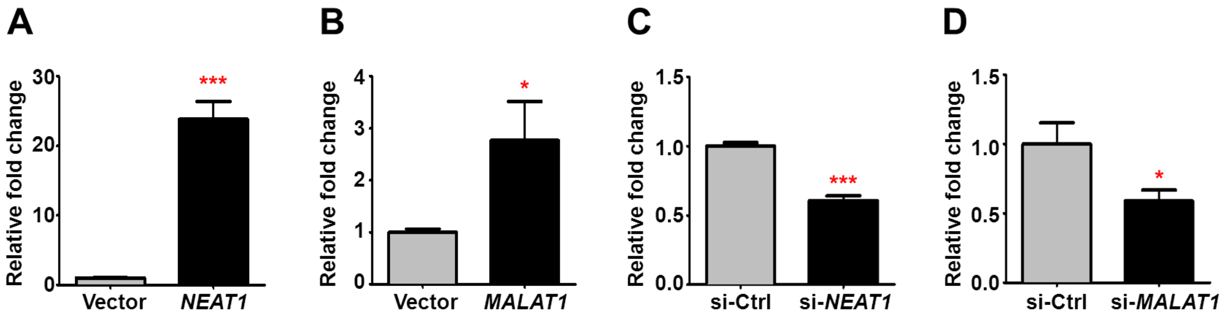

Figure S4 RNA expression level of the manipulated *NEAT1* and *MALAT1* in A549 lung cancer cells. A549 cells transfected with *NEAT1* expression vector (**A**) or *MALAT1* expression vector (**B**), si-*NEAT1* oligo (si-*NEAT1*) (**C**) or si-*MALAT1* oligo (si-*MALAT1*) (**D**) were harvested and subjected to qRT-PCR assays for *NEAT1* and *MALAT1* RNA expression. Data are mean ± SEM. *P*-values were determined by two-tailed Student's *t*-test. **P* < 0.05; ****P* < 0.001.

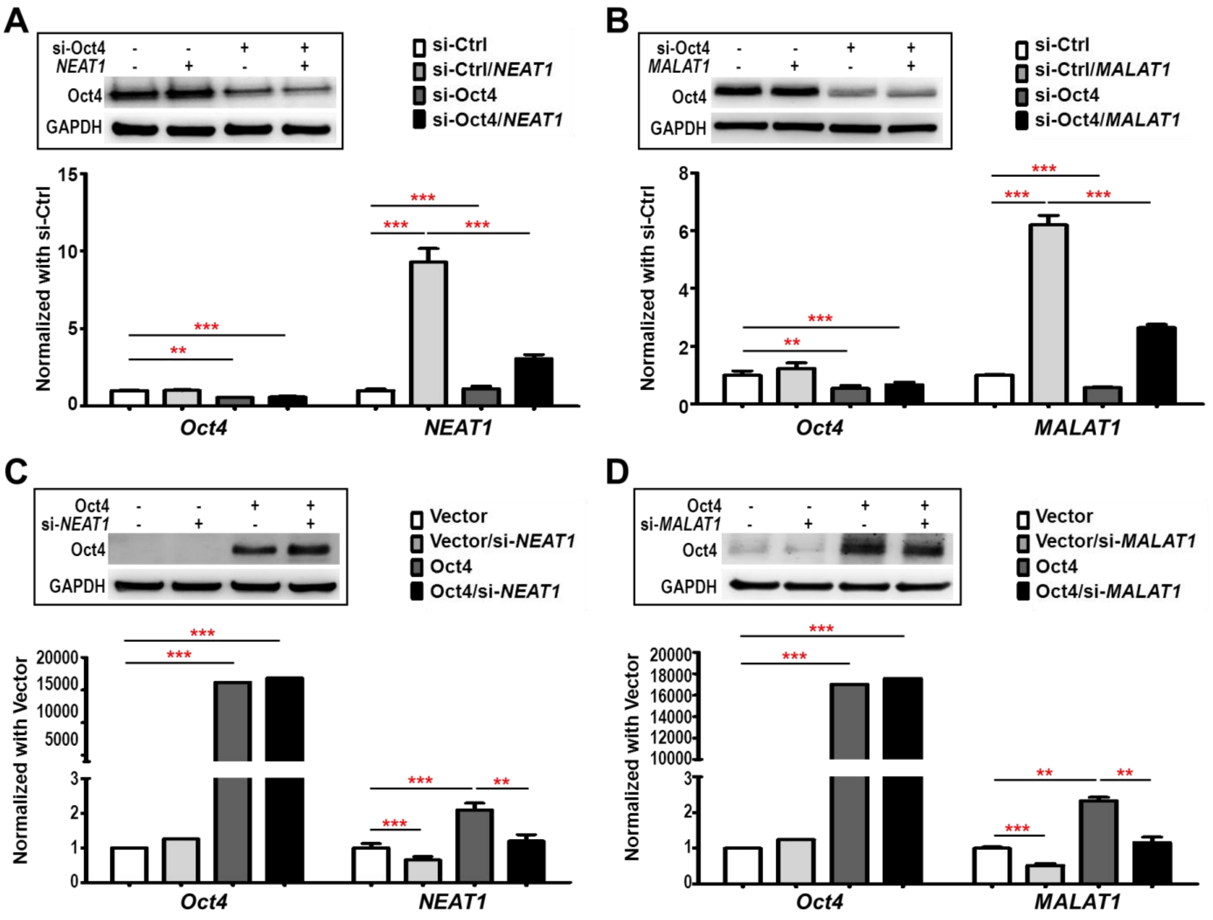

Figure S5 RNA and protein expression level of the manipulated Oct4, *NEAT1* and *MALAT1* in A549 lung cancer cells. A549 cells were transfected with expression vectors of *NEAT1* (**A**) or *MALAT1* (**B**) alone or together with si-Oct4 oligo (si-Oct4). A549 cells were transfected with si-*NEAT1* oligo (si-*NEAT1*) (**C**) or si-*MALAT1* oligo (si-*MALAT1*) (**D**) alone or together with Oct4 expression vector. Cell lysates were subjected to qRT-PCR assays for *Oct4*, *NEAT1* and *MALAT1* RNA expression or Western blot analysis for Oct4 protein expression (inset). GAPDH serves as an internal control. Data are mean ± SEM. *P*-values were determined by two-way ANOVA. ***P* < 0.01; ****P* < 0.001.
